# A Smartphone-Driven Acoustic Platform for Non-Invasive Modulation of Cellular Behavior in Microfluidic Channels

**DOI:** 10.3390/mi17030329

**Published:** 2026-03-06

**Authors:** Giulia Valenti, Emanuela Cutuli, Francesca Guarino, Maide Bucolo

**Affiliations:** 1 Department of Biomedical and Biotechnological Science, University of Catania, Via Santa Sofia 89, 95123 Catania, Italy; francesca.guarino@unict.it; 2 Department of Electrical Electronic and Computer Science Engineering, University of Catania, Via Santa Sofia 64, 95123 Catania, Italy; emanuela.cutuli@phd.unict.it (E.C.); maide.bucolo@unict.it (M.B.)

**Keywords:** microfluidics, acousto-mechanical perturbation, image processing

## Abstract

In recent years, passive cell manipulation in microfluidic devices has emerged as a crucial tool for biomedical and biotechnological applications, allowing control over cell positioning and behavior without the need for chemical labels or complex external forces. However, achieving precise and tunable modulation of cell dynamics remains a challenge, particularly with low-cost and non-invasive methods. In this work, we present a novel approach that leverages controlled acousto-mechanical perturbations (AMPs) to modulate cell arrangement and behavior in microchannels. By coupling a smartphone-driven audio speaker with a microfluidic device, acoustic signals are converted into mechanical vibrations of the tubing, generating AMPs that interact with hydrodynamically driven flows. Experiments with yeast cells and silica beads under different flow conditions revealed that acoustic stimulation induced periodic flow dynamics, with yeast cells showing tunable, flow-dependent responses while inert particles exhibited weak and stable modulation. Frequency-domain analysis highlighted a dominant response synchronized with the applied acoustic protocol, accompanied by higher-frequency components characteristic of acoustic actuation. These results demonstrate that simple, low-cost acoustic actuation revealed distinct dynamical responses between rigid inert particles and deformable biological cells and enable label-free cellular manipulation. The proposed platform offers a versatile, non-invasive, and accessible approach for controlled cell manipulation in microfluidics.

## 1. Introduction

Precise manipulation of cell populations at the microscale underpins modern biomedical innovations, from personalized diagnostics to the development of organ-on-a-chip platforms that simulate complex tissue environments [1]. These technologies allow researchers to probe cellular behavior in response to defined stimuli, guiding cell positioning, aggregation, or separation with high spatial resolution. However, existing methods for achieving controlled cellular arrangement and behavior often depend on expensive equipment, intricate experimental setups, and highly trained operators. This reliance on high-end instrumentation and complex protocols poses significant barriers to widespread adoption, especially in resource-limited settings, where scalable and cost-effective alternatives are urgently needed.

Applications of cellular manipulation span multiple domains. In tissue engineering, precise cell arrangement enables the development of scaffolds, organoids, and three-dimensional constructs that mimic native tissues [2,3]. In cancer biology, immunology, and microbiology, controlled microscale environments provide insight into aggregation dynamics, metastasis, immune activation, and biofilm formation [4,5]. In diagnostics, cellular manipulation supports efficient sorting of circulating tumor cells, immune subtypes, or blood fractions for downstream analyses [6]. Traditional approaches for cellular manipulation can be broadly categorized into passive and active methods. Among passive strategies, inertial microfluidics has emerged as a widely adopted solution. This technique operates without external actuation and exploits the intrinsic hydrodynamic forces that arise in laminar microscale flows. Cell separation is achieved through carefully designed channel geometries and fixed flow conditions, which induce size- and deformability-dependent inertial lift forces that passively drive particles toward equilibrium positions [7]. While effective, these systems rely strongly on precise channel design, often requiring sophisticated fabrication and limited adaptability to varying biological samples [8]. Micropatterning technologies represent another passive approach, in which predefined chemical or topographical features on substrates guide cell organization without dynamic control. Although powerful for tissue engineering and mechanobiology applications, these techniques typically depend on cleanroom fabrication, advanced imaging, and specialized expertise [9]. Active methods, in contrast, rely on externally applied fields to dynamically manipulate cells and particles. Among these, label-free techniques, including hydrodynamic, acoustic, and electrokinetic approaches, are particularly attractive, as they enable real-time control without the use of chemical or fluorescent labels, thereby preserving native cellular properties. In active hydrodynamic manipulation, channel geometries such as spirals, contractions, or expansions are combined with externally modulated flow conditions or time-dependent perturbations to dynamically reshape the flow field [10]. These configurations generate controllable lift forces, microvortices, and flow-focusing effects that actively confine, redirect, or trap cells, providing a higher degree of tunability compared to purely passive inertial systems [11]. Hydrodynamic stress is characterized by high physiological relevance, as it directly reproduces in vivo shear flow conditions [12]. Beyond purely hydrodynamic approaches, a range of complementary techniques have been developed to probe cellular mechanical and functional properties [13,14,15]. These could include mechanical and deformability-based methods [16,17,18,19], which investigate membrane stiffness, deformability, and viscoelasticity, as well as thermal and metabolic techniques [20,21], which provide information on oxygen consumption, glucose uptake, lactate production, and heat exchange. Although these multimodal strategies yield high-quality and detailed information, they typically require complex experimental setups, such as magnetic tweezers, force sensors, high-speed cameras, micro-heaters, and optical or electrochemical sensors, and sophisticated data analysis. Their microchip designs are also highly specialized: mechanical approaches often rely on tailored channel geometries with constrictions or micropillars, while thermal and metabolic systems integrate micro-heaters, temperature sensors, and metabolite detectors. Acoustic methods exploit sound waves to generate pressure fields that interact with cells in suspension and are commonly classified into bulk acoustic wave (BAW) and surface acoustic wave (SAW) platforms, where standing or surface-bound waves induce cell positioning and separation [22]. Acoustic stimulation enables highly tunable on-chip flow control through modulation of frequency, amplitude, and duty cycle, and has been widely applied to cell separation [23,24]. However, despite their versatility, these approaches exhibit moderate physiological relevance and require integrated transducers, precise flow control, temperature stabilization, and accurate alignment between acoustic fields and microfluidic channels. Electrokinetic approaches provide another active label-free category. Electrophoresis has been primarily used for cell classification [25], while dielectrophoresis (DEP) and electrorotation (ROT) extend interrogation to dielectric properties such as membrane capacitance and cytoplasmic conductivity. DEP applies translational forces in non-uniform electric fields, allowing manipulation and separation of large cell populations. It is particularly suited for high-throughput applications like cell sorting or viability discrimination, but provides limited dielectric characterization [26]. ROT, by contrast, applies torque in rotating fields, enabling detailed single-cell characterization of dielectric properties [27,28]. Its throughput, however, remains low, since analyses are restricted to individual cells. All the discussed label-free techniques are summarized in Table 1.

This study introduces a non-invasive and low-cost approach to modulate cell arrangement and behavior in microfluidic channels using controlled acousto-mechanical perturbations (AMP). Building upon recent work demonstrating the use of audio signals to generate programmable dynamic flow patterns in microfluidic systems [29], our approach extends this concept beyond fluid manipulation alone by explicitly incorporating and analyzing the response of suspended microparticles and biological cells. The system combines a smartphone-driven audio speaker with a microfluidic device, where acoustic tones induce mechanical vibrations in the tubing. These vibrations perturb the internal fluid flow and actively modulate microparticle dynamics, revealing distinct behaviors depending on their physical and biological properties. This hybrid acoustic–mechanical platform provides a portable, affordable, and user-friendly means not only for flow control but also for investigating microparticle dynamics. Video processing using Digital Particle Image Velocimetry (DPIV)-based algorithm [30,31,32,33] captures both time-dependent and spatially varying particle dynamics, while combined time- and frequency-domain analyses elucidate transient behaviors and spectral responses. Overall, the system offers several advantages, including low cost, portability, label-free operation, and flexible modulation of flow conditions. Key results indicate clear differentiation between inert and biological particles, demonstrating sensitivity to externally applied mechanical cues. Limitations include constraints on frequency range, hardware sensitivity, and particle size compatibility. The platform also shows potential applications in mechanobiology research, real-time monitoring of cellular responses, and exploratory studies for drug testing or diagnostics.

The paper is organized as follows. Section 1 describes materials and methods, including the microfluidic device, experimental setup, sample preparation, stimulation protocol, and experimental campaign. Section 2 presents the results: Section 2.1 focuses on low-frequency hydrodynamic responses (0–2 [Hz]), while Section 2.2 addresses higher-frequency responses (10–28 [Hz]).

## 2. Materials and Methods

This section provides a detailed explanation of the working principle and design of the microfluidic device, along with an overview of the sample preparation process. It also describes the experimental setup and protocol and the experimental campaign carried out to investigate the behavior of micro-particles under acousto-mechanical perturbation (AMP).

### 2.1. System Working Principle

This study presents a novel approach for generating controlled acousto-mechanical perturbations (AMPs) to reproducibly modulate fluid dynamics within a microfluidic system. Unlike conventional acoustofluidic platforms based on bulk acoustic wave (BAW) or surface acoustic wave (SAW) devices, which rely on acoustic radiation forces and standing wave formation within the fluid, the present system operates through predominantly mechanical perturbations arising from externally induced tubing vibrations directly transmitted to the microchannel. The proposed setup therefore combines conventional hydrodynamic actuation for driving the flow with an external acousto-mechanical module designed to introduce programmable mechanical perturbations.

**Figure 1:**
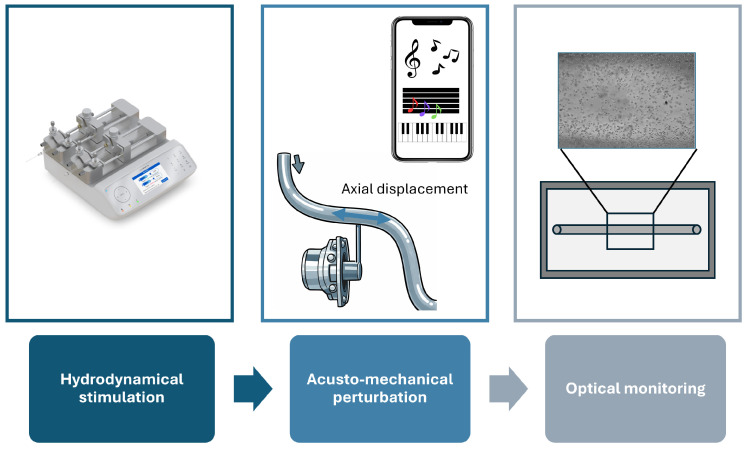
Schematic illustration of the working principle of the acousto-mechanical system. The system integrates a syringe pump for controlled flow injection, a smartphone-based audio signal generator, and an acoustic actuator that converts the audio signal into axial mechanical displacements of the tubing. A CAD rendering of the microfluidic device is also shown, highlighting the observation area in the microchannel where micro-particles motion is optically monitored.

As illustrated in Figure 1, the working principle of the system consists of a hydrodynamic actuation source provided by syringe pumps, which generate a steady and precisely controlled flow through the microfluidic channels. In parallel, a smartphone generates controlled audio signals that are amplified and used to drive a loudspeaker. The oscillatory motion of the speaker diaphragm is mechanically transmitted to the microfluidic tubing via an L-shaped shaft, ensuring direct coupling between the actuator and the fluidic circuit. This mechanical coupling converts the electrical driving signal into periodic mechanical deformations of the tubing, which propagate to the enclosed fluid and generate controlled perturbations of the flow field inside the microchannels, as described in detail in Section Acousto-Mechanical Transductor. As a result, the fluid experiences a superposition of steady hydrodynamic forcing and time-dependent acousto-mechanical modulation. These perturbations induce periodic displacements of suspended micro-particles, whose dynamics reflect the combined effects of flow rate, stimulation frequency, and mechanical coupling efficiency. The resulting particle motion is quantitatively characterized through high-resolution imaging and subsequent analysis of flow patterns and micro-particle trajectories, enabling the investigation of frequency-dependent and time-resolved dynamical responses.

### 2.2. Microfluidic Channel: Design and Realization

**Figure 2:**
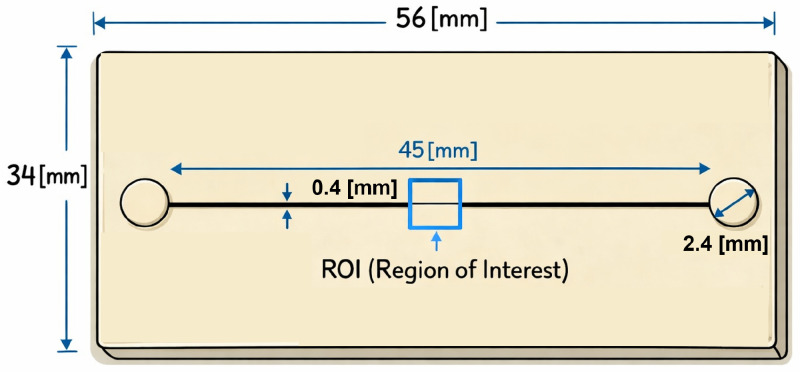
Schematic view of the microfluidic device geometry. The figure highlights the rectangular base and the linear microchannel, including the inlet and outlet regions, as well as the selected observation area located approximately at the midpoint of the channel, where particle motion is optically monitored.

The microchannel used in this study was fabricated using a master-slave approach based on 3D printing, detailed in [33,34]. In particular, the master was created via inkjet 3D printing, while the final microfluidic device was fabricated using polydimethylsiloxane (PDMS). The selected device, as shown in Figure 2, features a streamlined rectangular base designed to meet the requirements of the intended application. It incorporates a linear channel, 45 [mm] long and 0.40 [mm] wide, with an inlet and an outlet both having a uniform diameter of 2.4 [mm]. The base measures 56 [mm] in length and 34 [mm] in height, with sidewalls 1.5 [mm] thick. Additionally, Figure 2 shows the region of interest (ROI), i.e., the chosen observation area (180 × 140 pixel), located approximately at the midpoint of the channel, around 22 [mm] from the inlet.

### 2.3. Sample Preparation

Two distinct types of micro-particles were employed for the experimental plan: Silica beads and Eukaryotic Yeast cells of Saccharomyces cerevisiae. All their physical properties are described in Table 2. Silica beads were suspended in a water–glycerol solution composed of 80% glycerol and 20% water with a density of approximately 1200 kg/$m^{3}$. This specific mixture was selected to achieve a density closely matching that of the silica beads, thereby minimizing sedimentation and ensuring their buoyant suspension throughout the experiments. In contrast, yeast cells were suspended in a saline solution, specifically Phosphate Buffered Saline (PBS) with a density of 1126 [kg/m^3^], due to its physiological compatibility and effective buffering capacity. For both silica beads and yeast cells, the concentration of suspensions was carefully adjusted to $10^{8}$ micro-particles per 10 [mL] of solution.

### 2.4. Experimental Setup

**Figure 3:**
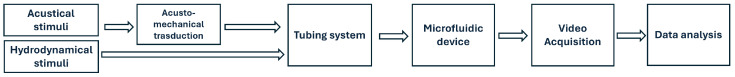
System design block scheme.

The experimental setup used to investigate the behavior of micro-particles within the microchannel under the influence of acousto-mechanical perturbation (AMP) is schematically represented in Figure 3.

The system combines acousto-mechanical perturbations and hydrodynamic stimuli, which are delivered to the microfluidic channel via a tubing system. In particular, the hydrodynamic flow, generated by Chemyx Fusion 4000 pumps (Chemyx Inc., Stafford, TX, USA), is influenced by acoustic perturbations that modulate its dynamics.

The microfluidic device is placed on the stage of a microscope (OPTIKA IM-300 Phi, Ponteranica, BG, Italy) equipped with a 10× objective lens, enabling high-resolution visualization. Imaging is achieved through a high-resolution CCD camera (340M Fast Frame, Thorlabs, Newton, NJ, USA), offering a resolution of 640×480 pixels (with a pixel size of 7.4 [μm]). The camera operates at a frame rate of 57 [FPS] with an exposure time of 17.438 [ms]. Data are transferred to a PC via USB and acquired using ThorCam™ software (version 3.7.0). Data analysis is performed on a PC featuring an Intel Core i7 processor, Intel Iris Xe Graphics, 16 GB of RAM, and a 512 GB SSD.

**Figure 4:**
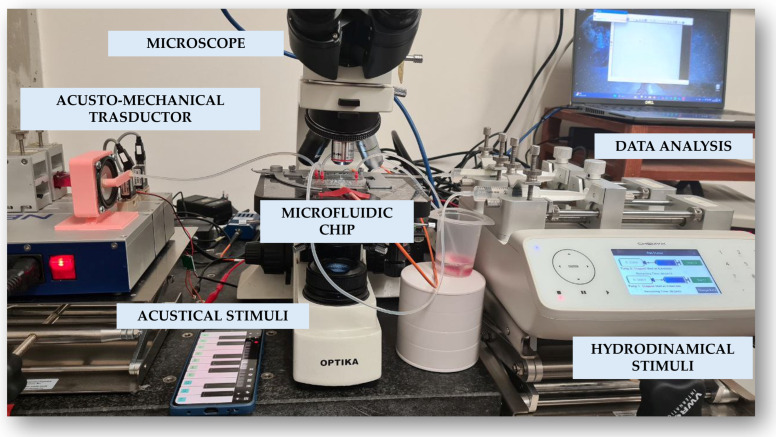
Complete Experimental Setup. The picture illustrates the integrated experimental system, which includes hydrodynamic actuation through syringe pumps and acoustic actuation driven by a smartphone. The setup also comprises an acousto-mechanical transducer coupled to the tubing and integrated within the microfluidic device. Finally, the optical microscope connected to a camera is shown; the camera records particle motion and is interfaced with a PC used for both video acquisition and subsequent data analysis.

This comprehensive setup provides precise actuation and observation capabilities, essential for accurate experimental outcomes. A picture of the real experimental setup is shown in Figure 4.

#### Acousto-Mechanical Transductor

**Figure 5:**
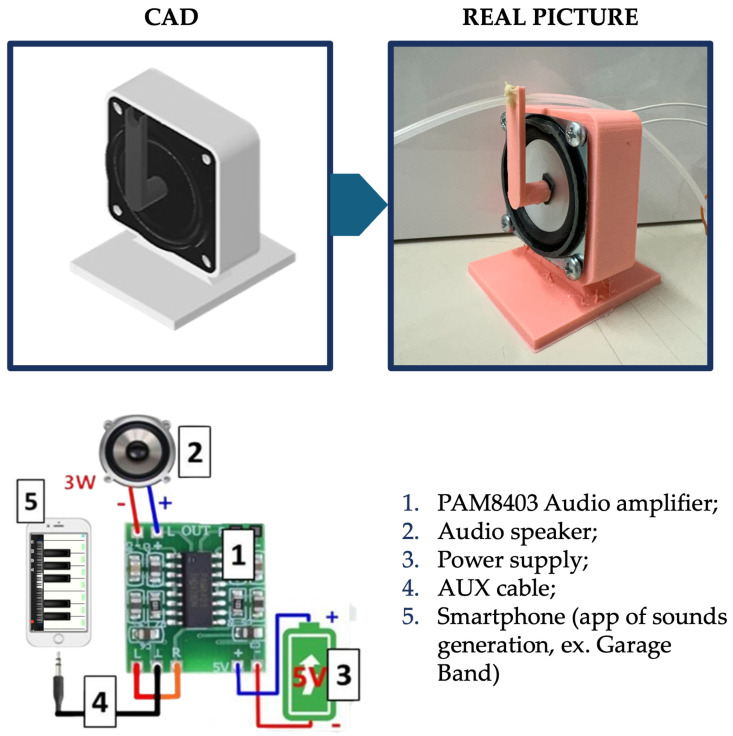
3 Watt speaker housed in the 3D-printed case, the PAM8403 audio amplifier, and the cable connections.

The acousto-mechanical perturbations (AMPs) are generated by a smartphone connected via an AUX cable to a PAM8403 audio amplifier. (PAM8403 Super Mini Digital Power Amplifier Board (2×3 W Class-D), ARCELI, China) The amplifier, powered by a dedicated 5 V supply, drives a 3 W loudspeaker which converts audio signals generated by the smartphone into mechanical vibrations. The loudspeaker is housed within a custom-designed enclosure fabricated using Autodesk^®^ Fusion 360 (Autodesk Fusion 360 (version 2.0), Autodesk, Inc., San Francisco, CA, USA) and 3D printing. The enclosure is specifically engineered to minimize parasitic mechanical vibrations and to ensure efficient and stable acoustic coupling with the microfluidic system. A key component of the setup is a 3D-printed L-shaped shaft mechanically coupled to the center of the speaker diaphragm, where the vibration amplitude is known to be maximal and most uniform before propagating radially across the membrane with increasing modal complexity at higher frequencies. This shaft acts as a mechanical transmission element, efficiently conveying the acoustic excitation from the loudspeaker to the fluidic system. The shaft is hollow, allowing the tubing to pass through its interior and directly connect to the microfluidic channel. In this configuration, the acoustic oscillations are effectively transferred to the tubing, thereby inducing controlled pressure and flow perturbations within the system. The complete assembly and its connections are illustrated in Figure 5.

### 2.5. Experimental Campaign and Protocol

In the experimental campaign carried out, two distinct and constant flow rates were investigated for silica beads: 0.0001 [mL/min] and 0.001 [mL/min]. For yeast cells, in addition to these same flow rates, a higher flow rate was also considered. Preliminary results indicated that the parameters extracted from data processing increased with flow rate for yeast cells; therefore, a flow rate of 0.005 [mL/min] was additionally investigated to confirm this behavior. In total, 10 experiments were conducted, encompassing various combinations of micro-particle types, flow rates, and vibrational conditions.

Each experimental flow condition was evaluated under two operational modes:

- **NO AMP** mode: reference condition without acousto-mechanical perturbations, used for system calibration and baseline measurements;
- **AMP** mode: condition in which acousto-mechanical perturbations were applied.

The acoustic excitation frequency was set at the G-sharp note, with perturbations applied at two octave levels, specifically the first and fourth octaves, corresponding to frequencies of 25.96 [Hz] and 415.30 [Hz], respectively. The entire experimental campaign is summarized in Table 3.

**Figure 6:**
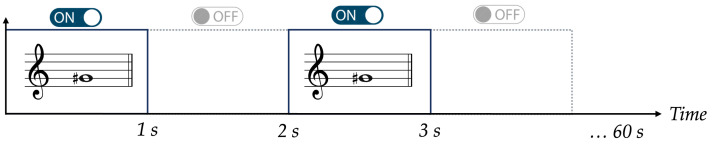
Protocol of experiments: a periodic ON–OFF scheme consisting of 1 s of acoustic stimulation (ON) alternated with 1 s of rest (OFF), repeated continuously over a total duration of 60 s.

The experimental protocol adopted for the AMP mode is described as follows. Acousto-mechanical perturbations were applied at regular one-second intervals according to a repeating pattern, as illustrated in Figure 6. Specifically, a periodic ON–OFF scheme was implemented, consisting of 1 s of stimulus application (ON phase) followed by 1 s without stimulus (OFF phase). This cycle was continuously repeated for the entire duration of the experiment, equal to 1 min.

### 2.6. DPIV-Based Algorithm

The video analysis was performed using a well-established and previously validated method based on Digital Particle Image Velocimetry (DPIV-based). This technique has been widely employed in earlier works to investigate microscale flow dynamics and micro-particles behavior, and its methodology has been described in detail elsewhere [30,31,32,33].

The DPIV-based algorithm enabled the non-invasive analysis of micro-particle motion within microfluidic environments by processing high-speed video recordings. By tracking the displacement of particles between consecutive frames, the algorithm computes velocity vectors, providing quantitative insight into the hydrodynamic responses of the particles. To refine spatial resolution, the DPIV analysis employs a three-step discrete Fourier transform (DFT) approach across multiple interrogation areas, generating time-resolved velocity vector maps that reveal the displacement of micro-particles within a designated region of interest (ROI).

A comprehensive study was conducted to evaluate the responses of these micro-particles under AMP mode in both the time and frequency domains.

Time-domain analysis enables the characterization of particle displacement over time. The key parameter extracted from this analysis is the *velocity range*, defined as the difference between the maximum and minimum values of the time-dependent, spatially averaged velocity components along the flow-parallel (*x*) and transverse (*y*) directions. Accordingly, the velocity range is expressed as:

(1) $$r_{{\bar{V}}_{i}\left(t\right)}=\max\;\left({\bar{V}}_{i}\left(t\right)\right)-\min\;\left({\bar{V}}_{i}\left(t\right)\right),\;i\in \left\{x,y\right\},$$

where ${\bar{V}}_{x}\left(t\right)$ and ${\bar{V}}_{y}\left(t\right)$ denote the longitudinal (parallel to the flow) and transverse (orthogonal to the flow) components of the spatially averaged velocity, respectively.

In parallel, frequency-domain analysis provided insight into the spectral components governing particle dynamics. Specifically, the Fourier spectra of the time-averaged velocity components along the longitudinal (*x*) and transverse (*y*) directions were computed and analyzed. From these spectra, the *maximum peak at the fundamental frequency* was identified as

(2) $$f_{p,i}=\max\left|{\bar{V}}_{i}\left(f\right)\right|,\;i\in \left\{x,y\right\},$$

where ${\bar{V}}_{i}\left(f\right)$ denotes the frequency spectrum of time-averaged velocity component along direction *i*.

These metrics quantify the interaction between the intrinsic micro-particles flow and the externally applied oscillatory stimulus, taking into account the particles’ biophysical properties.

Furthermore, specific frequency bands ranging from 10 [Hz] to 28 [Hz] were selected to investigate the intrinsic response of each micro-particle type to acoustic stimulation. More generally, for a given frequency interval $\left[f_{1},f_{2}\right]$, the overall spectral response has been quantified by integrating the Fourier-transformed velocity over the selected band, defined as

(3) $$A_{\left[f_{1},f_{2}\right]}=\sum_{f=f_{1}}^{f_{2}}{\bar{V}}_{x}\left(f\right),$$

## 3. Results and Discussion

### 3.1. Calibration of the Acousto-Mechanical Perturbation

To quantitatively assess the mechanical perturbation effectively delivered to the microchannel, additional calibration experiments were performed by measuring the time-resolved outlet flow rate of the microfluidic device using a commercial flow sensor (SF04 Chip, Sensirion, Stäfa, Switzerland). In a hydrodynamically driven microfluidic system, flow-rate oscillations directly reflect inlet pressure pulsations; therefore, these measurements provide a reliable quantitative proxy of the mechanical excitation transmitted from the actuator to the microchannel.

During the calibration experiments, the same protocol described in Section 2.5 was applied. Measurements were conducted using PBS as the working fluid, without suspended particles, in order to isolate the purely hydrodynamic response of the system. A steady baseline flow rate of 0.001 mL/min was imposed using syringe pumps, while cyclic acousto-mechanical perturbations were generated by the transducer described in this Section. The stimulation consisted of a periodic sequence of 1 s ON followed by 1 s OFF, continuously applied for 1 min. The driving signal corresponded to the G# note at the first and fourth octave, matching the experimental conditions used in the cell and particle experiments reported in this study.

**Figure 7:**
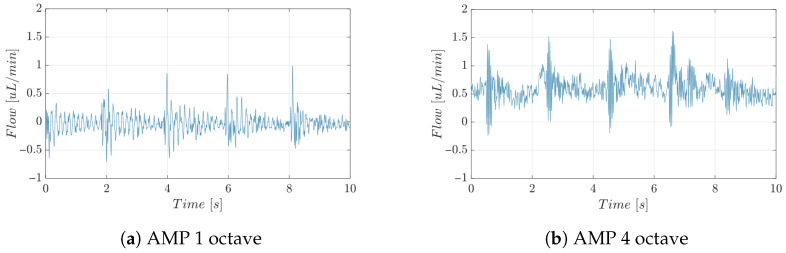
Representative 10 [s] segment of the measured flow-rate signal during calibration experiments under cyclic acousto-mechanical stimulation. A steady hydrodynamic flow of 0.001 [mL/min] was imposed, while the stimulation protocol consisted of alternating 1 [s] ON (G# note) and 1 [s] OFF phases applied continuously for 1 min. Figure (**a**) corresponds to stimulation at the first octave, and Figure (**b**) corresponds to stimulation at the fourth octave.

Figure 7 shows representative 10 s segments of the measured flow-rate signals for both excitation frequencies. A clear and reproducible periodic modulation of the flow rate is observed, synchronized with the ON–OFF stimulation phases. In particular, a systematic increase in flow rate occurs during the ON phases, confirming that the applied driving waveform produces a measurable pressure perturbation effectively transmitted to the microchannel. Notably, the amplitude of the induced flow-rate modulation strongly depends on the acoustic excitation frequency. In particular, stimulation at the fourth octave produces a larger flow-rate increase compared to the first octave. In addition, at the higher excitation frequency the flow signal does not fully relax back to the baseline value during the OFF phase, suggesting the presence of a residual mechanical excitation. This behavior is consistent with a stronger mechanical coupling between the acoustic transducer and the tubing–microfluidic system at higher frequencies, resulting in larger and more persistent pressure pulsations at the inlet of the microchannel. Overall, these calibration measurements establish a direct quantitative link between the actuator input parameters and the resulting hydrodynamic perturbation within the microchannel.

### 3.2. Micro-Particles Hydrodynamic Response in the 0–2 Hz Band

**Figure 8:**
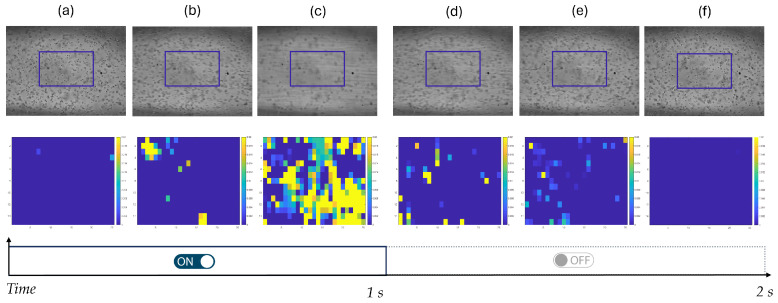
Dynamic behavior of yeast cells during acoustic stimulation of G sharp in $4^{\circ }$ octave and a flow rate of 0.001 [mL/min], illustrated through sequential frames and corresponding velocity distribution maps in the range 0–0.02 [mm/s]. (**a**) Initial condition after switching on the acoustic excitation. (**b**) Onset of particle motion. (**c**) Maximum dynamical response under sustained acoustic excitation. (**d**) Early decay phase after switching off the acoustic stimulus. (**e**) Further relaxation of the system. (**f**) Final quiescent state.

A comprehensive observation of the hydrodynamic response of micro-particles under acousto-mechanical perturbation is provided through a representative experiment, as depicted in Figure 8.

This figure illustrates the experimental protocol described in Section 2.5 through a representative sequence of frames showing the dynamic response of yeast cells to alternating phases of acoustic excitation (ON) and rest (OFF). For each frame, the corresponding velocity field within the selected region of interest (ROI) is reported, with a color scale ranging from 0 to 0.02 [mm/s].

This sequence of representations highlights the direct response of both the fluid velocity field and yeast cell motion to the applied acoustic excitation. At the onset of acoustic excitation, although the stimulus is already switched ON, yeast cells initially remain stationary and uniformly distributed within the ROI, and the corresponding velocity field exhibits values close to zero across the entire area (see Figure 8a). This indicates that the mechanical perturbation has just been initiated and that the system has not yet developed a measurable hydrodynamic response. As the stimulation proceeds (see Figure 8b), the first detectable particle displacements emerge, manifested as localized regions of non-zero velocity in the corresponding velocity map, marking the transition from a quiescent state to an actively driven regime. Under sustained acoustic excitation (Figure 8c), the system reaches its maximum dynamical response, characterized by pronounced collective motion of the yeast cells and by velocity magnitudes approaching approximately 0.02 [mm/s] over an extended portion of the ROI. When the acoustic stimulus is switched OFF, the externally applied mechanical forcing is removed and particle motion progressively decreases. The velocity field correspondingly shows a gradual reduction in magnitude, indicating the transition from forced to passive hydrodynamic conditions (see Figure 8d). This decay phase continues until yeast cells progressively slow down and the velocity field approaches near-zero values across most of the ROI, reflecting the relaxation of the system following the interruption of acoustic excitation (see Figure 8e). At the end, the system returns to a quiescent state in which yeast cells appear again stationary and randomly distributed, and the velocity field becomes uniformly close to zero, confirming the reversible and controllable nature of the acousto-mechanically induced dynamics (see Figure 8f).

In the following discussion, a detailed analysis of the results obtained for silica beads and yeast cells is presented. Two complementary analyses were performed in distinct spectral bands in order to highlight the different dynamical responses of the micro-particles to the applied stimulation. Within the 0–2 [Hz] frequency band, the analysis focused on two key metrics previously defined: the velocity range $r_{{\bar{V}}_{x}\left(t\right)}$, extracted from the time-domain velocity signal, and the dominant spectral peak $f_{p}$, identified from the Fourier spectra of the time-averaged velocity components (see Section 2.6). In the second spectral band, spanning 10–28 [Hz], a distinct and unexpected response was observed. To further investigate this behavior, the integral-based metric $A_{\left[10-28\;\mathrm{Hz}\right]}$, introduced in the frequency-domain analysis, was evaluated under all experimental conditions, considering all imposed flow rates.

For all three quantitative parameters considered in the study, velocity range $r_{{\bar{V}}_{x}\left(t\right)}$, the dominant spectral peak $f_{p}$ and the integral-based metric $A_{\left[10-28\;\mathrm{Hz}\right]}$ are reported as the mean of three independent replicates (n=3). The associated variability was quantified by the standard error of the mean (SEM), calculated as $\mathrm{SEM}=\mathrm{STD}/\sqrt{3}$, enabling a statistically robust and consistent comparison between the responses of silica beads and yeast cells under the different experimental conditions.

**Figure 9:**
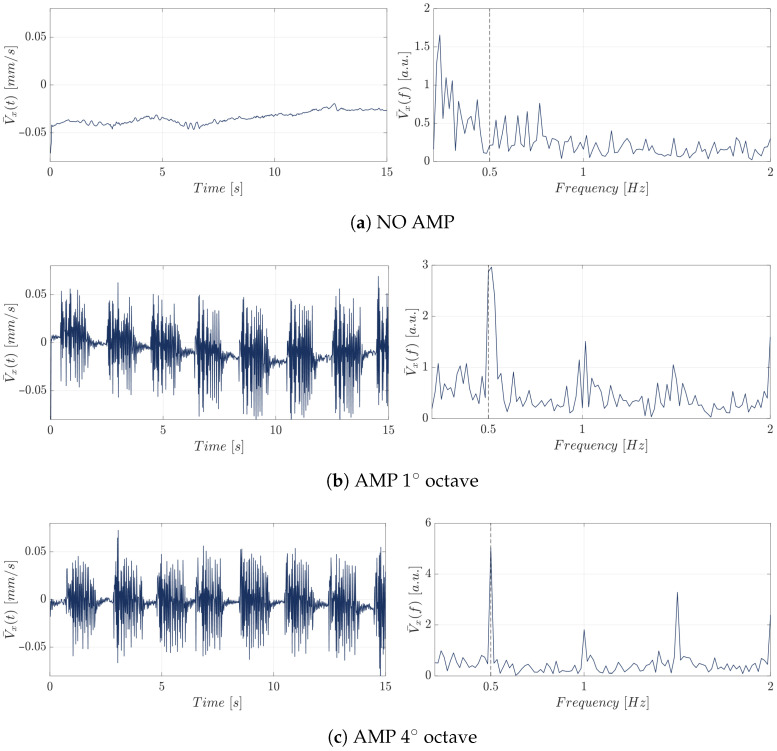
Time-domain velocity signal (left panels) and corresponding frequency spectrum (0–2 [Hz]) (right panels) for silica beads at a flow rate of 0.0001 [mL/min] under different acousto-mechanical perturbation (AMP) modes: (**a**) NO AMP, (**b**) AMP first octave and (**c**) AMP fourth octave.

The first set of experiments investigated silica beads to assess their hydrodynamic response to acousto-mechanical perturbation. Representative time-domain responses and the corresponding spectra (0–2 [Hz] band) for silica beads at flow rate of 0.0001 [mL/min] are shown in Figure 9.

For sake of brevity, only the velocity trend and spectra corresponding to the flow rate condition of 0.0001 [mL/min] are reported in Figure 9. Additional data are available from the authors upon request. However, similar patterns were observed for the other flow rates analyzed, confirming the consistency of the findings across different experimental conditions. For both stimulated conditions, i.e., AMP $1^{\circ }$ octave (see Figure 9a) and AMP $4^{\circ }$ octave (see Figure 9b), a prominent peak was observed at 0.5 [Hz], matching the imposed stimulation frequency corresponding to the protocol. This peak was absent in the control condition without stimulation, confirming its origin in the acoustic excitation protocol.

**Figure 10:**
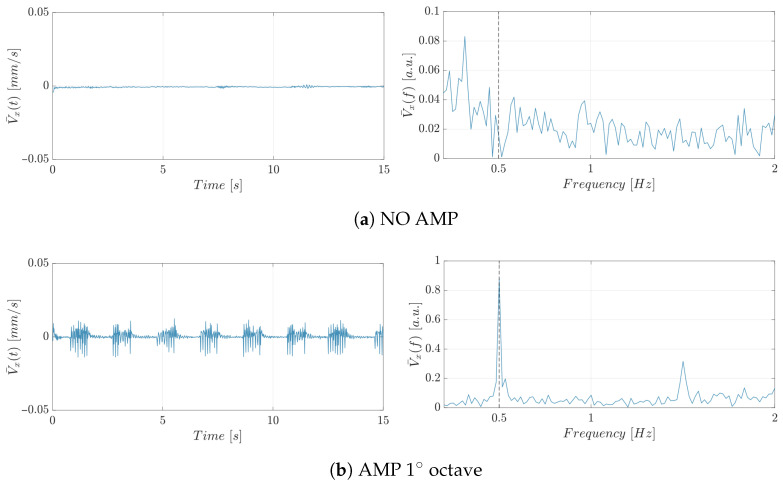

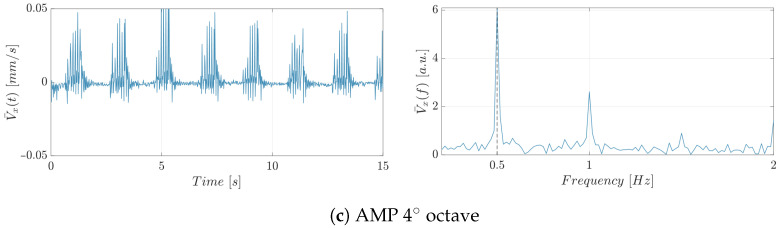
Time-domain velocity signal (left panels) and corresponding frequency spectra (0–2 [Hz]) (right panels) for yeast cells at a flow rate of 0.0001 [mL/min] under different acoustic stimulation conditions: (**a**) NO AMP, (**b**) AMP first octave and (**c**) AMP fourth octave.

Following the experiments on silica beads, yeast cells were analyzed to assess their hydrodynamic response under identical acoustic stimulation conditions. The experimental setup was kept consistent with that used for silica beads, enabling direct comparison between the two micro-particles type. Figure 10 illustrates their time-domain behavior and corresponding frequency spectra (0–2 [Hz] band) in the experimental condition with flow rate of 0.0001 [mL/min] under different acousto-mechanical perturbation (AMP) mode. In the frequency domain, the same dominant peak, dictated by the stimulation protocol, was consistently observed when the acoustic stimulus was applied.

**Figure 11:**
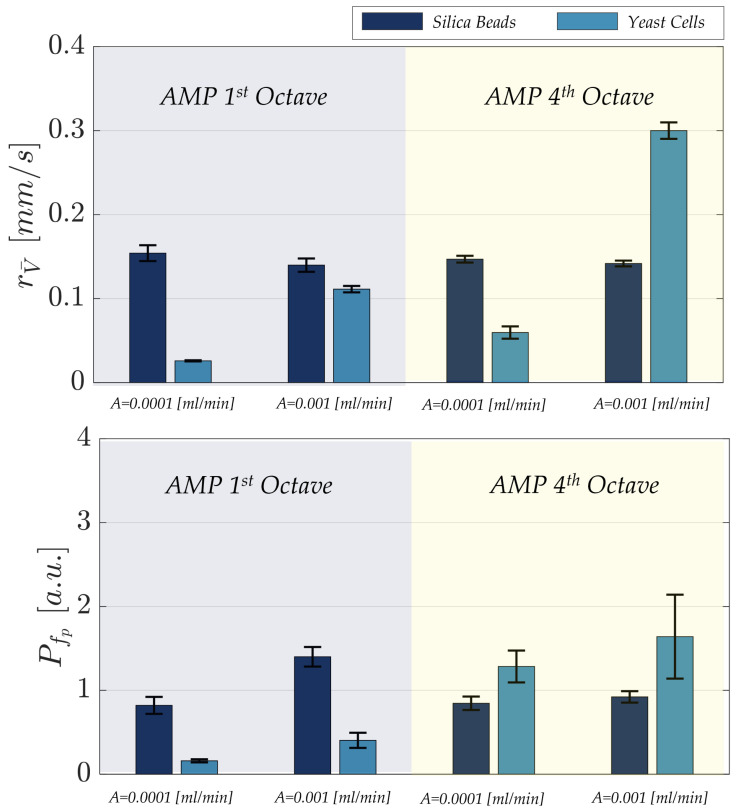
Bar plots of velocity range $r_{\bar{V}}\left(t\right)$ and amplitude of the frequency spectrum peak $f_{p}$ for silica beads and yeast cells measured at the first and fourth octaves (AMP 1 octave and AMP 4 octave) and at two flow rates (0.0001 [mL/min] and 0.001 [mL/min]). Data are reported as the mean of three independent experiments (n=3), and error bars represent the standard error of the mean (SEM).

The amplitude of the frequency peak varied significantly across different acoustic stimuli, specifically between first- and fourth-octave excitations, and between particle types. These variations indicate distinct micro-particle responses to the applied perturbation. To quantify and compare the acoustic response of silica beads and yeast cells under different flow rates and stimulation protocols, bars plot representations of the extracted metrics was employed. Figure 11 illustrates the values of velocity range and peak of dominant frequency for both micro-particle types across all experimental conditions: NO AMP, AMP under first- and four-octave stimulations for both flow rates: 0.0001 [mL/min] and 0.001 [mL/min].

As illustrated in the comparative bar plots (Figure 11), a clear contrast emerges between the behavior of silica beads and yeast cells under varying experimental conditions. For silica beads, the velocity range remains largely stable, varying only slightly between 0.165 and 0.176 [mm/s] across all flow rates (0.0001 and 0.001 [mL/min]) and acoustic stimulations (first- and fourth-octave), while the dominant frequency peak increases moderately from 3 to 5. In contrast, yeast cells exhibit pronounced changes: the velocity range increases from 0.032 [mm/s] to 0.121 [mm/s] under first-octave stimulation, and from 0.073 [mm/s] to 0.18 [mm/s] under fourth-octave stimulation, as the flow rate rises from 0.0001 to 0.001 [mL/min]. Similarly, the dominant frequency peak of yeast cells increases from 1 to 3 for first-octave stimulation and from 6 to 8 for fourth-octave stimulation, highlighting a strong dependence on both flow rate and acoustic stimulation. These results indicate that yeast cells are more sensitive to the applied perturbations, whereas silica beads maintain a stable and consistent velocity profile across the tested conditions.

Observing the distinct behaviors of yeast cells in response to acoustic stimulation at flow rates of 0.0001 [mL/min] and 0.001 [mL/min], we deemed it essential to investigate their response at a higher flow rate of 0.005 [mL/min].

**Figure 12:**
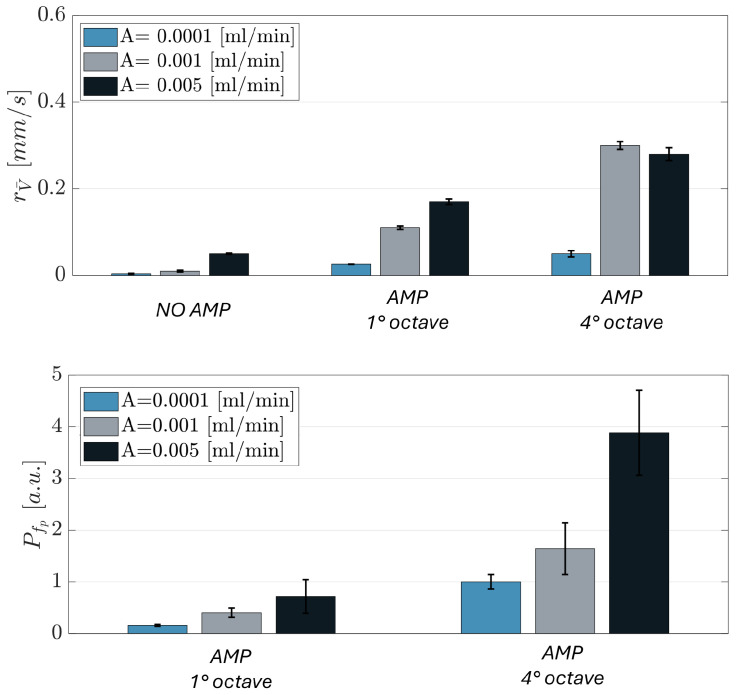
Bar plot of velocity range $r_{\bar{V}}\left(t\right)$ and amplitude of the frequency spectrum peak $f_{p}$ for yeast cells under different acoustic stimulation (AMP 1 octave and AMP 4 octave) at three flow rates: 0.0001, 0.001 and 0.005 [mL/min]. Data are reported as the mean of three independent experiments (n=3), and error bars represent the standard error of the mean (SEM).

This additional condition aimed to evaluate whether the observed trends, specifically the increases in velocity range and peak frequency amplitude, remained consistent or exhibited new dynamics under enhanced flow conditions. As illustrated in the comparative histograms in Figure 12, analysis across the three flow rates reveals a clear and consistent trand: both velocity range and peak frequency amplitude systematically increase with rising flow rates and corresponding octave shifts.

In conclusion, these findings emphasize the stark contrast between silica beads and yeast cells in their responsiveness to acoustic stimulation. The silica beads exhibit a rigid and unvarying behavior across different conditions, demonstrating their limited capacity for modulation through acoustic inputs. In contrast, the results clearly demonstrate that the behavior of yeast cells can be actively modulated by tuning the acoustic and mechanical conditions.

### 3.3. Micro-Particle Hydrodynamic Response in the 10–28 Hz Band

A detailed frequency-domain analysis of the system response in the 10–28 [Hz] range reveals a clear modification of the dynamical behavior of suspended micro-particles when acousto-mechanical perturbations are applied, both along the longitudinal (x) and transverse (y) directions of the microchannel.

**Figure 13:**
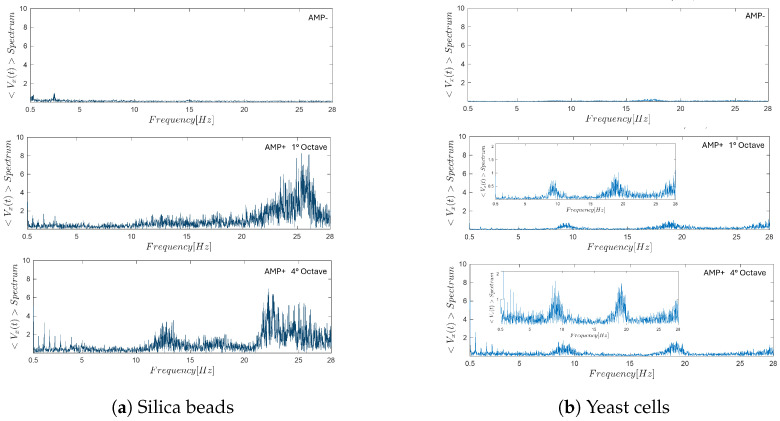
Frequency-domain response of micro-particles under different acoustic stimulation conditions at a flow rate of 0.0001 [mL/min], with all spectra displayed up to 28 [Hz]. The top row shows hydrodynamic behavior without acousto-mechanical perturbation (NO AMP), the middle row corresponds to the response to the G-sharp tone in the first octave (AMP 1 octave), and the bottom row shows the response to the fourth octave stimulation (AMP 4 octave).

Figure 13a,b report the velocity spectra obtained for silica beads and yeast cells, respectively, under three different experimental conditions: absence of acoustic stimulation (NO AMP), stimulation at the G-sharp note in the first octave (AMP 1st octave), and stimulation at the fourth octave (AMP 4th octave. In the NO AMP condition (top rows of Figure 13a,b), the spectra remain essentially flat over the entire investigated frequency range, indicating the absence of dominant frequency components and confirming that particle motion is governed solely by steady hydrodynamic forcing without any periodic modulation. When acoustic stimulation is applied at the first octave (middle rows), a marked spectral reorganization is observed. In Figure 13a, silica beads exhibit the emergence of well-defined peaks at specific frequencies, corresponding to the imposed acoustic excitation and its harmonics. These peaks indicate a strong coupling between the external acoustic signal and the induced particle dynamics. A similar behavior is observed for yeast cells in Figure 13b, although with lower spectral amplitude, suggesting a weaker but still detectable sensitivity of biological particles to the acousto-mechanical perturbation. Under fourth-octave stimulation (bottom rows), the frequency response becomes even more structured. For silica beads (Figure 13a), multiple pronounced peaks appear over a broader frequency interval, reflecting an enhanced and more complex dynamical response to higher-frequency acoustic forcing. Yeast cells (Figure 13b) also display clear frequency-dependent features, with identifiable peaks that mirror those observed for silica beads, albeit with reduced magnitude and increased spectral broadening.

An extensive analysis was performed on the area $A_{\left[10-28\;\mathrm{Hz}\right]}$, defined as the integral of the spectral velocity curve over a selected frequency interval (see Section 2.6). Specifically, the bands $A_{\left[10-15\;\mathrm{Hz}\right]}$, $A_{\left[18-23\;\mathrm{Hz}\right]}$ and $A_{\left[23-28\;\mathrm{Hz}\right]}$ were investigated.

**Figure 14:**
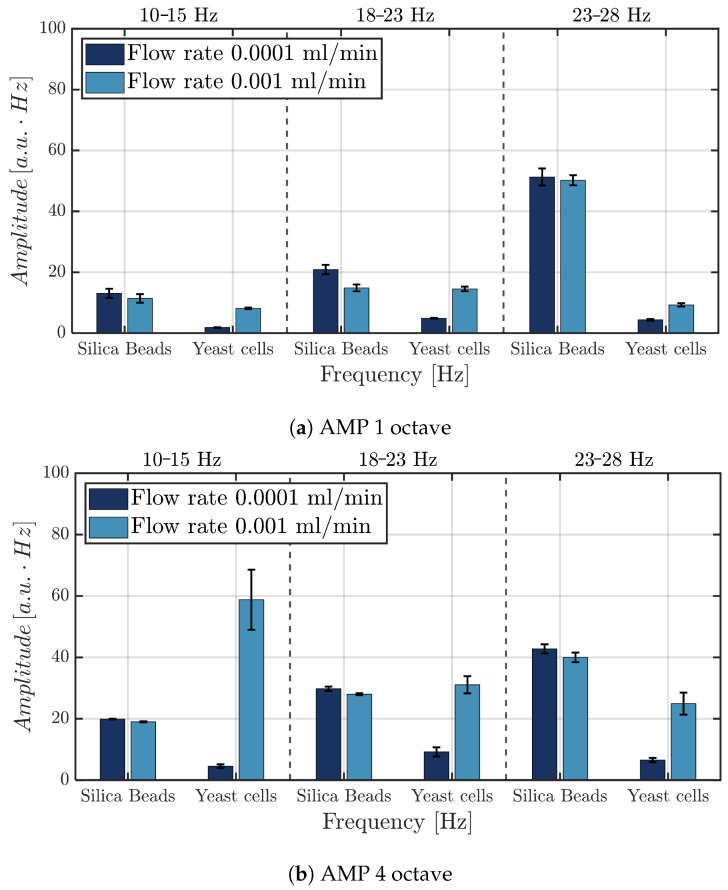
Integral analysis of the longitudinal (x-direction) spectral response. The figure reports the values of the spectral area computed as the integral of the velocity spectrum in three different bands (10–15 [Hz], 18–23 [Hz], and 23–28 [Hz]), for silica beads and yeast cells under NO AMP and AMP conditions. Results are shown for (**a**) first-octave and (**b**) fourth-octave stimulation at different flow rates. Values are expressed as the mean of three independent experiments (n=3), and error bars indicate the standard error of the mean (SEM).

The results of the longitudinal x-directional analysis are summarized in Figure 14. As observed, the spectral behavior of silica beads and yeast cells differs markedly in the first and fourth octaves. For silica beads, increasing the flow rate led to a reduction in the integral of the spectral curve, whereas for yeast cells, the integral increased with higher flow rates. This contrast highlights distinct dynamic responses of the two types of micro-particles when subjected to acoustic stimulation.

In addition to the longitudinal analysis, a complementary investigation was performed along the transverse (y) direction, perpendicular to the main flow axis. Since the hydrodynamic stream within the microfluidic channel was perturbed by the acousto-mechanical stimuli applied at the inlet, it was hypothesized that such perturbations could also generate transverse flow components.

**Figure 15:**
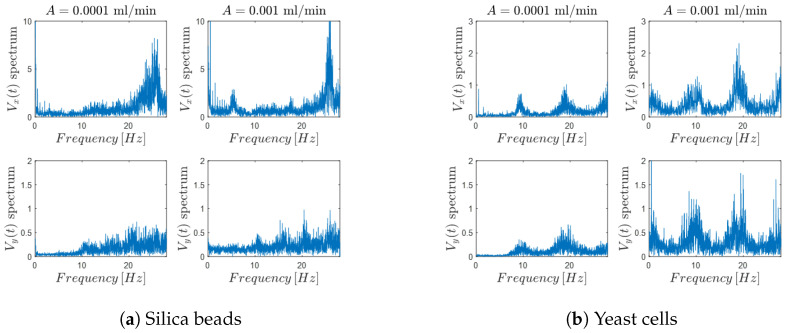
Transverse (y-direction) frequency response under acoustic stimulation. Frequency spectra of the mean velocity components for (**a**) silica beads and (**b**) yeast cells under AMP conditions. In each panel, the top row shows the longitudinal (x-direction) spectral response, while the bottom row reports the corresponding transverse (y-direction) spectra, enabling direct comparison between the two flow components.

The spectra in y-axis is presented in Figure 15 for both silica bead suspensions (Figure 15a) and yeast cell suspensions (Figure 15b), together with the corresponding spectra of the longitudinal (x) velocity component shown for direct comparison. While the silica beads do not exhibit a clear or systematic transverse spectral response, yeast cells display distinct frequency behavior in the y-direction that closely match those observed along the x-axis. Although these transverse behaviour is characterized by lower amplitude and increased noise, their recurrence within the same frequency bands as the longitudinal spectra confirms the presence of acoustically induced transverse flow components.

This comparative analysis indicates that the transverse dynamic response is specific to yeast cells and is not systematically observed in inert silica particles, suggesting that the phenomenon is linked to the biological or mechanical properties of the cells. Overall, these results demonstrate that acoustic perturbation modifies the flow field in both spatial directions for yeast, emphasizing the robustness and spatial extent of the response in biologically active systems.

## 4. Conclusions and Future Developments

In this work, a low-cost, non-invasive acoustic platform capable of modulating and probing micro-particles dynamics within microfluidic environments through smartphone-driven acousto-mechanical perturbations was adopted. This approach offers a potentially accessible and flexible alternative to conventional, equipment-intensive systems, providing a basis for exploring how micro-particles respond to controlled mechanical cues. By combining time-domain metrics, such as the velocity range, with frequency-domain descriptors, including the dominant spectral peak and the integral-based spectral area over selected frequency bands, the platform enabled a clear discrimination between inert silica beads and living yeast cells. While silica beads exhibited stable and largely invariant responses across flow rates and stimulation protocols, yeast cells showed pronounced, stimulation-dependent variations, highlighting their sensitivity to externally applied mechanical cues and intrinsic biophysical properties. These results demonstrate that the proposed platform is not only suitable for delivering acoustic stimulation, but also effective in capturing differential mechanical responses in a label-free manner. This capability provides a foundation for exploring how controlled acousto-mechanical perturbations influence cellular dynamics, transport, and potentially intercellular interactions within confined microfluidic environments. Beyond fundamental investigations, the platform holds promise for a range of potential applications. In diagnostics, real-time monitoring of cellular responses to mechanical stimulation could, in principle, support label-free assessments of phenotypic alterations, such as variations in stiffness or activation states. In drug testing, the system may enable exploratory studies of how pharmacological treatments influence cellular dynamics and intercellular signaling within mechanically controlled microenvironments, parameters that are often overlooked in standard assays.

Future developments will focus on expanding the operational capabilities of the proposed platform to further enhance its versatility and performance. In particular, extending the accessible frequency range and improving hardware sensitivity will enable the investigation of a broader spectrum of particle sizes and cell types, supporting more comprehensive mechanobiological studies. Optimization of stimulation protocols and refinement of detection strategies will contribute to increased robustness and discrimination power across different experimental conditions. In parallel, the integration of microcontrollers and programmable control electronics will allow the implementation of automated, repeatable, and precisely timed excitation protocols. This evolution will preserve the accessibility of smartphone-driven actuation while enabling higher temporal resolution and improved experimental reproducibility. Overall, through continued technological refinement and system integration, the platform is expected to evolve into a flexible and scalable experimental tool for probing cellular mechanobiology, with strong potential to support advanced applications in both research and translational contexts.

## Figures and Tables

**Table 1:** Summary of label-free techniques for cell manipulation.

Technique	Working Principle	Advantages	Disadvantages	References
Inertial microfluidics	Exploits size- and deformability-dependent inertial lift forces in laminar flows	No external actuation; high-throughput	Requires precise channel design; limited adaptability	[7,8]
Micropatterning	Chemical/topographical cues guide cell organization	High spatial resolution; useful for tissue engineering	Cleanroom fabrication; specialized expertise	[9]
Hydrodynamic manipulation	Modulated flows and channel geometries generate lift forces and microvortices	Real-time dynamic control; physiological relevance;	Complex flow modulation; specialized microchannels	[10,11,12]
Acoustic (BAW/SAW)	Sound waves generate pressure fields affecting cell positions	Tunable; non-invasive	Moderate physiological relevance; requires transducers and alignment	[22,23,24]
Dielectrophoresis (DEP)	Non-uniform electric fields apply translational forces based on dielectric properties	High-throughput cell sorting;	Limited single-cell dielectric characterization	[26]
Electrorotation (ROT)	Rotating electric fields apply torque to probe dielectric properties	Detailed single-cell analysis	Low throughput; restricted to individual cells	[27,28]
Mechanical/deformability-based	Constrictions, micropillars, or external forces probe stiffness and viscoelasticity	Insight into mechanical properties	Requires sensors, tweezers, imaging; complex setups	[16,17,18,19]
Thermal/metabolic	Measures oxygen consumption, heat, and metabolite fluxes	Functional cellular insights	Integration of heaters and sensors; complex analysis	[20,21]

**Table 2:** Physical properties of micro-particles.

Micro-Particles Type	Mass [kg]	Radius [m]	Density [kg/m^3^]
Silica Beads	$1.36\times 10^{-13}$	$3.0\times 10^{-6}$	1200
Yeast Cells	$7.37\times 10^{-14}$	$2.5\times 10^{-6}$	1126

**Table 3:** Summary of the experimental conditions, including flow rates, acousto-mechanical perturbation modes, and excitation frequencies for the investigated micro-particles.

Micro-Particles Type	Flow Rate [mL/min]	AMP Mode	Octaves in AMP Mode
Silica Beads	0.0001, 0.001	NO AMP/AMP	$1^{\circ }$ octave (25.96 Hz),
Yeast Cells	0.0001, 0.001, 0.005		$4^{\circ }$ octave (415.30 Hz)

## Data Availability

Data will be made available on request.
